# Propensity Score-Based Approaches in High Dimension for Pharmacovigilance Signal Detection: an Empirical Comparison on the French Spontaneous Reporting Database

**DOI:** 10.3389/fphar.2018.01010

**Published:** 2018-09-18

**Authors:** Émeline Courtois, Antoine Pariente, Francesco Salvo, Étienne Volatier, Pascale Tubert-Bitter, Ismaïl Ahmed

**Affiliations:** ^1^Biostatistics, Biomathematics, Pharmacoepidemiology and Infectious Diseases, INSERM, UVSQ (Université Paris-Saclay), Institut Pasteur, Villejuif, France; ^2^Bordeaux Population Health Research Center, Pharmacoepidemiology Team (UMR 1219), INSERM, University of Bordeaux, Bordeaux, France

**Keywords:** pharmacovigilance, signal detection, propensity score in high dimension, spontaneous reports, penalized multiple regression, FDR

## Abstract

Classical methods used for signal detection in pharmacovigilance rely on disproportionality analysis of counts aggregating spontaneous reports of a given adverse drug reaction. In recent years, alternative methods have been proposed to analyze individual spontaneous reports such as penalized multiple logistic regression approaches. These approaches address some well-known biases resulting from disproportionality methods. However, while penalization accounts for computational constraints due to high-dimensional data, it raises the issue of determining the regularization parameter and eventually that of an error-controlling decision rule. We present a new automated signal detection strategy for pharmacovigilance systems, based on propensity scores (PS) in high dimension. PSs are increasingly used to assess a given association with high-dimensional observational healthcare databases in accounting for confusion bias. Our main aim was to develop a method having the same advantages as multiple regression approaches in dealing with bias, while relying on the statistical multiple comparison framework as regards decision thresholds, by considering false discovery rate (FDR)-based decision rules. We investigate four PS estimation methods in high dimension: a gradient tree boosting (GTB) algorithm from machine-learning and three variable selection algorithms. For each (drug, adverse event) pair, the PS is then applied as adjustment covariate or by using two kinds of weighting: inverse proportional treatment weighting and matching weights. The different versions of the new approach were compared to a univariate approach, which is a disproportionality method, and to two penalized multiple logistic regression approaches, directly applied on spontaneous reporting data. Performance was assessed through an empirical comparative study conducted on a reference signal set in the French national pharmacovigilance database (2000–2016) that was recently proposed for drug-induced liver injury. Multiple regression approaches performed better in detecting true positives and false positives. Nonetheless, the performances of the PS-based methods using matching weights was very similar to that of multiple regression and better than with the univariate approach. In addition to being able to control FDR statistical errors, the proposed PS-based strategy is an interesting alternative to multiple regression approaches.

## 1. Introduction

Once a drug is introduced on the market, many people are exposed to it in real-life conditions, which can be very different from those evaluated in clinical trials. The goal of pharmacovigilance is to detect as quickly as possible potential adverse reactions which could be induced by drug exposure. To achieve this challenging task, health authorities collect and monitor spontaneous reports of suspected adverse events (AEs), mainly from practitioners. In France, such a pharmacovigilance database is maintained by the National Agency for the Safety of Drugs and Health Products (*Agence Nationale de Sécurité du Médicament et des Produits de Santé*, ANSM). It contained around 431,000 reports at the end of July 2016. In recent years, about 36,000 reports have been reported annually.

To exploit this amount of data, several automated signal detection tools have been developed. The term signal refers to a (drug, AE) pair whose association is highlighted by a signal detection method. The aim of such methods is to draw attention to unexpected associations by acting as hypothesis generators. The signals thus generated must be further investigated by pharmacovigilance experts in order to draw definite conclusions. The most common methods used by health agencies are disproportionality methods (Almenoff et al., 2007). These methods rely on a three-step strategy: for each (drug, AE) pair (1) determine the number of reports involving this specific pair; (2) construct a measure of the degree to which the observed count of reports exceeds the expected count if independence applies (the disproportionality measure); and (3) decide that a signal has occurred if this measure exceeds a specified threshold value. The procedure is conducted for all (drug, AE) pairs simultaneously (Ahmed et al., 2015). More recently, these disproportionality methods were extended by integrating a false discovery rate (FDR) estimation procedure in order to take into account comparison multiplicity (Benjamini and Hochberg, 1995; Ahmed et al., 2010).

To address the shortcomings of disproportionality methods in dealing with confounding like masking effect and co-prescription bias (Harpaz et al., 2012), multiple logistic regression approaches have been proposed. While being more computationally intensive, they have been shown through empirical studies to be promising signal detection methods (Caster et al., 2010; Harpaz et al., 2013; Marbac et al., 2016; Ahmed et al., 2018). Instead of considering aggregated data as disproportionality methods do, these newer approaches analyse spontaneous reports directly: the observation becomes the individual report, the outcome is the presence/absence of a given AE, and the covariates are all drug presence indicators (Caster et al., 2010). Because of the very large number of potential covariates, a lasso version of the logistic regression has been used (Tibshirani, 1996). It consists in maximizing the log-likelihood of a classical multiple logistic regression model, penalized by a function proportional to the *L*_1_ norm of the regression coefficients. This penalty makes it possible to shrink some coefficients associated with the covariates to exactly zero. Cross validation is commonly used to choose the penalty value and it achieves good performances in terms of prediction. However, determining the best penalization parameter in the variable selection framework is a challenging task.

Another way to deal with confounding bias issues is to summarize all the information into a propensity score (PS). PSs are classically used in epidemiological studies investigating one targeted association (i.e., one drug, one AE) to deal with confounding like indication bias. In recent years, this methodology has been used with high-dimensional health-care databases, like claims data, by collapsing all the numerous covariates present in these databases into a single value which is easier to manipulate (Seeger et al., 2005; Schneeweiss et al., 2009). Tatonetti et al. (2012) investigated the use of one PS strategy in the context of spontaneous reporting data and illustrated its interest by comparing it to a disproportionality method.

In this paper, we develop several PS-based approaches for signal detection from spontaneous reporting and compare them to penalized multiple regression approaches. We built the PSs with four different algorithms adapted to high dimensional settings and for each of them, we considered three different integration strategies to perform signal detection. Our proposed PS-based methods include a signal detection rule relying on FDR estimation. This comparison is performed with the French national pharmacovigilance database (2000–2016) using a large and recently published reference set pertaining to a common adverse reaction: drug-induced liver injury (DILI) (Chen et al., 2011, 2016).

## 2. Methods

### 2.1. Reference methods

The most common proposed methods for signal detection are disproportionality methods, which rely on the aggregated form of the data. These methods aim to detect higher-than-expected combinations of drugs and events in the database. Nevertheless, two kinds of biases are created by the univariate feature of disproportionality methods: masking and the co-reporting confounding effect (Harpaz et al., 2012). In order to address these two bias issues, other approaches in pharmacovigilance have lately emerged that rely on multiple logistic regressions (Caster et al., 2010). Individual spontaneous reports are directly analyzed instead of aggregated counts of drug-event combinations. We denote by *I* the total number of drugs and *J* the total number of AEs in the database. Let *Y*_*j*_ indicate the presence or absence of AE_*j*_, and *X*_*i*_ denote the presence or absence of drug_*i*_. The corresponding logistic model for each AE_*j*_ is

(1) $$\text{logit(Pr}(Y_{j}=1))=\beta_{0}^{j}+\sum_{i\;=1}^{I}\beta_{i}^{j}X_{i}.$$

In spontaneous reporting databases, the number of drug covariates *I* is very large. To deal with this high-dimensional issue and in order to derive the most parsimonious model, a penalized lasso logistic regression was implemented (Tibshirani, 1996). This consists in maximizing the log-likelihood of model (1) minus a penalization term

(2) $$\begin{array}{r} pen_{j}\left(\lambda \right)=\lambda \;|\beta^{j}{|}_{1}=\lambda \overset{I}{\underset{i\;=1}{\sum }}|\beta_{i}^{j}|. \end{array}$$

Depending on the penalization parameter λ, the lasso regression shrinks most of the coefficients associated to the covariates to exactly zero, so these covariates are not retained in the model. Usually cross validation is used to select the best value of λ in terms of prediction error, but this does not achieve good performance in a variable selection framework which is that of signal detection. Here, we considered two alternative strategies to this penalization parameter selection: the BIC-Lasso and the class-imbalance subsampling lasso (CISL) (Ahmed et al., 2018).

#### 2.1.1. BIC-lasso

This method uses the Bayesian Information Criterion. First, a lasso regression is computed for a predefined set of penalization parameter values λ_*k*_s. To each tested λ_*k*_ is associated a set of variables selected by the lasso regression. The BIC is then calculated from a classical logistic regression model for each of these sets:

(3) $$\begin{array}{r} \text{BIC}=-2\text{ln}\left(L\right)+k\text{ln}\left(N\right) \end{array}$$

where *L* is the likelihood of the regression model, *N* the number of observations, and *k* the number of parameters in the model. We declared as signals all drugs positively associated with the outcome in the model that minimizes the BIC.

#### 2.1.2. CISL

Another way to get around the penalization parameter selection issue is the stability selection method (Meinshausen and Bühlmann, 2010). To take into account the sparsity of pharmacovigilance databases with low frequencies of the outcomes, Ahmed et al. (2018) proposed a variation of this method: the CISL algorithm. In this method, samples are drawn following an nonequiprobable sampling scheme with replacement to allow a better representation of individuals who experienced the outcome of interest. For a given set of penalization parameter values, lasso logistic regressions are computed in each of these samples. The CISL procedure consists in computing the quantity:

(4) $$\begin{array}{r} {\hat{\pi }}_{i}^{b}=\frac{1}{E}\overset{E}{\underset{\eta \;=1}{\sum }}1\left[{\hat{\beta }}_{i}^{\eta ,b}>0\right], \end{array}$$

where *E* is the maximum value of covariates selected by all the lasso regressions, η∈{1, .., *E*} is the number of predictors selected and ${\hat{\beta }}_{i}^{\eta ,b}$ is the regression coefficient estimated by the logistic lasso for drug *i*, on sample *b*∈{1, .., *B*}, for a model including η covariates. An empirical distribution of ${\hat{\pi }}_{i}^{b}$ for each drug is obtained over all *B* samples. A covariate is finally selected by the CISL method if a given quantile of the distribution of ${\hat{\pi }}_{i}^{b}$ is non-zero. In this work, we considered the covariate sets established with the 5% and the 10% quantiles of these distributions.

In the following we refer to these two methods as the multiple regression approaches.

### 2.2. Propensity score approaches

The PS is defined as the probability of being exposed to a treatment given the observed covariates (Rosenbaum and Rubin, 1983). Conditionally on the PS, treatment exposure and the observed covariates are independent: patients with similar PSs will have on average similar covariate distributions between the exposed and unexposed subjects. This relies on the strong assumption that there are no unmeasured confounders. This means that all variables that affect both treatment assignment and outcome, thus potentially inducing spurious associations, have been measured. The PS is used in the context of observational studies as a balancing score that allows for non-randomized trials to reproduce the conditions of a randomized experiment by making observed baseline characteristics comparable in two different treatment groups. Thus, it addresses confounding biases like indication bias. In this framework, the PS is unknown and has to be estimated from observed data, usually using a logistic regression. Each patient is then assigned a predicted probability of being exposed that is calculated from the PS regression.

#### 2.2.1. Propensity score modeling

It is recommended to include predictors and confounders in the PS model i.e., covariates that are related to the outcome or to the outcome and the exposure. It is also strongly advised to avoid instrumental variables i.e., variables that are only related to the exposure (Patorno et al., 2013). In practice, it is sometimes recommended to make *a priori* variable selection according to expert knowledge (Brookhart et al., 2006). In the high-dimensional framework such as health-care databases where thousands of covariates are entered, this approach cannot hold. The high-dimensional propensity score (hdPS) algorithm was proposed to deal with this kind of difficulty (Schneeweiss et al., 2009). For a given exposure, the choice of candidate covariates to include in the PS model relies on empirical assessment of covariates prevalence and strength of association with the exposure and with the outcome of interest. The algorithm ranks all input variables by their potential for confounding and the investigator must choose the top-ranked variables to be included in the model for estimating the PS.

In the present work we aimed at computing the PS for each drug in the database by selecting among all the other drugs those to be included in the PS estimation model. We implemented four PS-estimation methods. We considered three variable selection algorithms: hdPS and the two other variable selection methods described above: the BIC-Lasso and the CISL [with the positive constraint for β replaced by a non-zero constraint in Equation (4)]. Using the BIC-lasso methodology, each covariate associated with the smallest BIC models was selected. Using CISL, all covariates with a 10% percentile of its distribution greater than zero were selected. Using hdPS, the 20 top-ranked variables were selected. For the sake of completeness, we also implemented a PS-estimation method which relies on a machine-learning algorithm, as was recently proposed by Lee et al. (2010): we considered a gradient tree boosting (GTB) algorithm (Hastie et al., 2009).

#### 2.2.2. Use of the propensity score

Once the PS is estimated, four methodologies may be used to remove confounding in estimating the treatment effect: (1) adjustment on the PS; (2) stratification on the PS; (3) matching on the PS; (4) weighting on the PS. Adjustment on the PS consists in considering the estimated PS as a covariate and including it as an additional variable, with the exposure, in the regression model on the outcome. Stratification on the PS is based on a population stratification according to the quantiles of the PS distribution. The treatment effect is then estimated in each subgroup of patients. Matching on the PS consists in matching one treated subject to one (or more) untreated subject which have similar values of their estimated PS. Classically, subjects are matched with a nearest neighbor matching algorithm within a specified caliper distance equal to 0.2 of the standard deviation of the estimated PS. Weighting on the PS amounts to assigning to each subject a weight that is calculated from the estimated PS. The treatment effect is estimated on the pseudo population which is built according to these weights (Austin, 2011).

In this study, we considered two of these four different PS methodologies. First we implemented an adjustment on the PS. For every (AE_*j*_, drug_*i*_) pair of interest, we computed the following logistic regression model:

(5) $$\text{logit(Pr}(Y_{j}=1))=\beta_{0}^{j}+\beta_{i}^{j}X_{i}+{\tilde{\beta }}_{i}^{j}{\hat{e}}_{i}$$

where ${\hat{e}}_{i}$ is the estimated PS of drug_*i*_ for all the observations.

We also implemented the inverse probability of treatment weighting (IPTW, Austin, 2011). For a given drug_*i*_, the weights to be attributed to each observation are defined by

(6) $$\begin{array}{r} w_{i}^{IPTW}=\frac{X_{i}}{{\hat{e}}_{i}}+\frac{1-X_{i}}{1-{\hat{e}}_{i}}. \end{array}$$

Because some drugs have been reported very rarely, and do not have many reports in common with AEs, matching subjects on PS may cause dramatic losses in terms of subjects who experienced both the exposure and the outcome. Keeping this constraint in mind, we performed another kind of weighting to mimic the classical matching by targeting the same estimand as pair-matching on the PS. The proposed weights are referred to the matching weights (MW) (Li and Greene, 2013; Franklin et al., 2017).

(7) $$\begin{array}{r} w_{i}^{MW}=\frac{\text{min}\left({\hat{e}}_{i},1-{\hat{e}}_{i}\right)}{X_{i}{\hat{e}}_{i}+\left(1-X_{i}\right)\left(1-{\hat{e}}_{i}\right)}. \end{array}$$

For these two weighting approaches, we then computed univariate weighted logistic regressions by considering $w_{i}^{IPTW}$ or $w_{i}^{MW}$.

For all these PS-based approaches, the signal detection rule is the one applied in the hypothesis testing framework. To take the multiplicity of the tested hypotheses into account, we applied an FDR controlling procedure adapted to the one-sided null hypothesis setting that stands in pharmacovigilance (Ahmed et al., 2010). An FDR adjusted *p*-value is estimated for each comparison tested. A signal is considered to occur if this *p*-value corrected for multiplicity is below an FDR threshold.

## 3. Material and comparison settings

To assess the performances of our PS-based approaches, we compared their ability to correctly classify true and false signals to multiple regression approaches presented above and a univariate approach, using the French pharmacovigilance database on a recently proposed reference set pertaining to DILI adverse events.

### 3.1. Comparison set-up

We compared the ability of each method to detect true signals and not to detect false signals. For the BIC-Lasso approach, we declare as signals all drugs positively associated with the outcome in the model that minimizes the BIC. All drugs selected by CISL are considered as a signal since the positive association with the outcome is taken into account in the algorithm. For all PS-based approaches, a 5% level FDR controlling procedure was used to determine which drug-AE associations are considered as signals. We also included a method based on a simple univariate logistic regression: for each AE_*j*_, *j*∈{1, .., *J*} and each drug_*i*_, *i*∈{1, .., *I*} we computed:

(8) $$\text{logit(Pr}(Y_{j}=1))=\beta_{0}^{j}+\beta_{i}^{j}X_{i}.$$

This method was implemented to serve as a reference level. It can be assimilated to a reporting odds ratio method, which is a disproportionality method for signal detection (van Puijenbroek et al., 2002). We applied the same FDR controlling procedure to this univariate approach.

All the analyses were performed with R version 3.4.0 (R Core Team, 2017). All the logistic regressions were computed with the speedglm R package v0.3–2. All lasso regressions were implemented using the glmnet R package v2.0–10. GTB algorithms for PS estimation were implemented with the xgb.train function from the xgboost R package 0.6–4. The default values of the xgb.train function were used, except for the learning rate, which was fixed at 0.1.

Table 1 summarizes the main advantages and disadvantages of the different signal detection methods presented in this section. For the sake of clarity, we refer to the multiple regression approaches in the following as BIC-Lasso, CISL-5%, CISL-10% and to the univariate approach as Univ. The terms adjustPS-BIC, mwPS-BIC, iptwPS-BIC, adjustPS-CISL, mwPS-CISL, iptwPS-CISL, adjustPS-GTB, mwPS-GTB, iptwPS-GTB, and adjustPS-hdPS, mwPS-hdPS, iptwPS-hdPS refer to the PS approaches implemented, the way PS was estimated (BIC, CISL, GTB or hdPS) and how PS was taken into account (adjustment, MW, or IPTW).

**Table 1:** Main advantages and disadvantages of the compared signal detection methods.

**Methods**	**Advantages**	**Disadvantages**
Univariate approach (disproportionality method)	Very fast computation time	Do not account for multiple
	Detection threshold based on classical test theory (*p*-values, FDR correction)	exposures
Penalized multiple logistic regression methods	Fast computation time	Detection threshold not relying on
	Account for multiple exposures	classical test theory
Propensity-score based methods	Account for multiple exposures	Long calculation time for
	Detection threshold based on classical test theory (*p*-values, FDR correction)	the propensity score estimation step

### 3.2. The french pharmacovigilance database

The performances of our approaches were evaluated and compared by an empirical analysis using data from the French pharmacovigilance database. Drugs are coded according to the 5th level of the Anatomical Therapeutic Chemical (ATC) hierarchy, and the AEs to the Preferred Term (PT) level of the Medical Dictionary for Regulatory Activities (MedDRA).

We applied some filters to the data and considered (i) only products which are known to be a drug (leaving out vaccines, phytotherapy, homeotherapy, dietary supplement, oligotherapy and enzyme inhibitor); (ii) reactions which are targeted as AEs (overdoses or medication errors are not taken into account). Drugs are listed according to their active substances, which are encoded with the ATC classification and additional codes for substances that do not have a corresponding code. We extracted data over the period 2000–2016, which represents 382,484 reports with 5,906 different AEs and 2,344 different drugs.

### 3.3. Set of reference signals

The set of reference signals that we used to compare the performances of the methods is the DILIrank set recently established by Chen et al. (2011) which considers a specific adverse event: DILI. After determining a list of keywords related to the DILI event, this set was developed by text-mining the FDA-approved drug labels. A severity level was associated to each keyword. Drugs were classified into three DILI-related categories according to where keywords appear in the labeling section (Warnings & Precautions section, for example) of the FDA-approved drug labels, and the severity level of the involved keywords: most DILI concern, less DILI concern and no DILI concern. In 2016, this classification was refined by including information from other data sources to assess the causal relationship between each considered drug and a DILI event. Only drugs confirmed as DILI cause were retained (Chen et al., 2016).

We translated DILI labeling keywords into PT codes from the MedDRA classification: 91 PT codes were considered as DILI related. Each spontaneous report involving one of these 91 PT was thus considered as a reported DILI event, which translated into 22,440 DILI reports in the French pharmacovigilance database. To avoid numerical issues due to very low drug-reporting frequency, we limited the comparison to drugs which have more than three reports in common with a DILI, and have more than ten reports in total. This restriction applied, we considered 922 different drugs out of the 2,344 original drugs from the spontaneous reporting database. Considering these drugs, the final DILIrank set accounted for 417 (drug, DILI) pairs: 90 no DILI concern pairs, 213 less DILI concern pairs and 114 most DILI concern pairs. We considered as negative controls the no DILI concern pairs, and as positive controls the most DILI concern ones, which involve drugs associated with severe DILI outcomes.

## 4. Results

Performances of the methods were assessed in terms of number of signals detected, number of true positives and number of false negatives identified, sensitivity and specificity, positive predictive value (PPV) and false discovery proportion (FDP). Table 2 summarizes the results. AdjustPS-BIC, adjustPS-CISL, adjustPS-GTB, and adjustPS-hdPS detected more signals than the other PS-based approaches with 308, 275, 273, and 310 signals respectively. All the iptwPS-based approaches led to the lowest number of signals with 35, 63, 70, 34 signals for iptwPS-BIC, iptwPS-CISL, iptwPS-GTB, and iptwPS-hdPS. The mwPS-based approaches gave an intermediate number of detected signals with 147, 121, 136, and 139 signals for mwPS-BIC, mwPS-CISL, mwPS-GTB, and mwPS-hdPS respectively. Multiple regression approaches detected between 99 and 173 signals. The univariate approach detected the largest number of signals: 359.

**Table 2:** Number of signals detected for each method.

**Method**	**Number of**	**Number of signals**	**True positives signals**			**False positives signals**		
	**generated signals**	**with known status**
		**(positive or negative)**

			*n*	**PPV**	**Sensitivity**	*n*	**FDP**	**Specificity**
Univ	359	105	86	81.90	75.44	19	18.10	78.89
BIC-lasso	173	66	64	96.97	56.14	2	3.03	97.78
CISL-5%	99	43	43	100.00	37.72	0	0.00	100.00
CISL-10%	109	48	48	100.00	42.11	0	0.00	100.00
adjustPS-BIC	308	96	81	84.38	71.05	15	15.62	83.33
mwPS-BIC	147	53	52	98.11	45.61	1	1.89	98.89
iptwPS-BIC	35	14	13	92.86	11.40	1	7.14	98.89
adjustPS-CISL	275	86	75	87.21	65.79	11	12.79	87.78
mwPS-CISL	121	50	49	98.00	42.98	1	2.00	98.89
iptwPS-CISL	63	17	14	82.35	12.28	3	17.65	96.67
adjustPS-GTB	273	85	74	87.06	64.91	11	12.94	87.78
mwPS-GTB	136	52	49	94.23	42.98	3	5.77	96.67
iptwPS-GTB	70	28	25	89.29	21.93	3	10.71	96.67
adjustPS-hdPS	310	93	83	89.25	72.81	10	10.75	88.89
mwPS-hdPS	139	54	53	98.15	46.49	1	1.85	98.89
iptwPS-hdPS	34	16	15	93.75	13.16	1	6.25	98.89

*For the BIC-lasso a signal is a positive association in the selected model, for CISL-5% and CISL-10% a signal is a selected variable obtained according to a distribution quantile (5% or 10%) in the CISL methodology. For Univ and all the PS-based approaches, a signal is an association FDR significant. The number of true positives signals is the number of positives controls detected by the method. The number of false positives signals is the number of negatives controls detected by the method*.

*PPV: Positive Predictive Value*.

*FDP: False Discovery Proportion*.

Multiple regression approaches gave the highest positive predictive values (PPV): 96.97, 100, and 100% for BIC-Lasso, CISL-5% and CISL-10% respectively. All the mwPS-based approaches gave similar results with a PPV around 98%, except for mwPS-GTB with a PPV equal to 94.23%. The univariate approach had the lowest PPV: 81.90%. AdjustPS-based approaches had slightly better performances: 84.38, 87.21, 87.06, and 89.25% for adjustPS-BIC, adjustPS-CISL, adjustPS-GTB, and adjustPS-hdPS respectively. Depending on the method used to estimate the PS, iptwPS-based approaches performed differently with PPVs ranging from 82.35% for iptwPS-CISL to 93.75% for iptwPS-hdPS.

Univ, adjustPS-BIC, adjustPS-CISL, adjustPS-GTB, and adjustPS-hdPS had the best performance in terms of sensitivity: between 64.91% for adjustPS-GTB and 75.44% for Univ, but they had the poorest specificity: between 78.9% for Univ and 88.9% for adjsutPS-hdPS. On the other hand, multiple regression approaches and mwPS-based approaches had very good specificity. BIC-Lasso, CISL-5% and CISL-10% had a specificity equal to 96.97, 100, and 100% respectively. MwPS-BIC, mwPS-CISL, mwPS-GTB, and mwPS-hdPS had a specificity equal to 98.11, 98.00, 94.23, and 98.15% respectively. Multiple regression approaches had sensitivity between 37.72% for CISL-5% and 56.14% for BIC-Lasso. MwPS-based approaches had sensitivity between 42.98% for mwPS-CISL and mwPS-GTB, and 46.49% for mwPS-hdPS. IptwPS-based approaches had good specificity but very low sensitivity.

**Figure 1:**
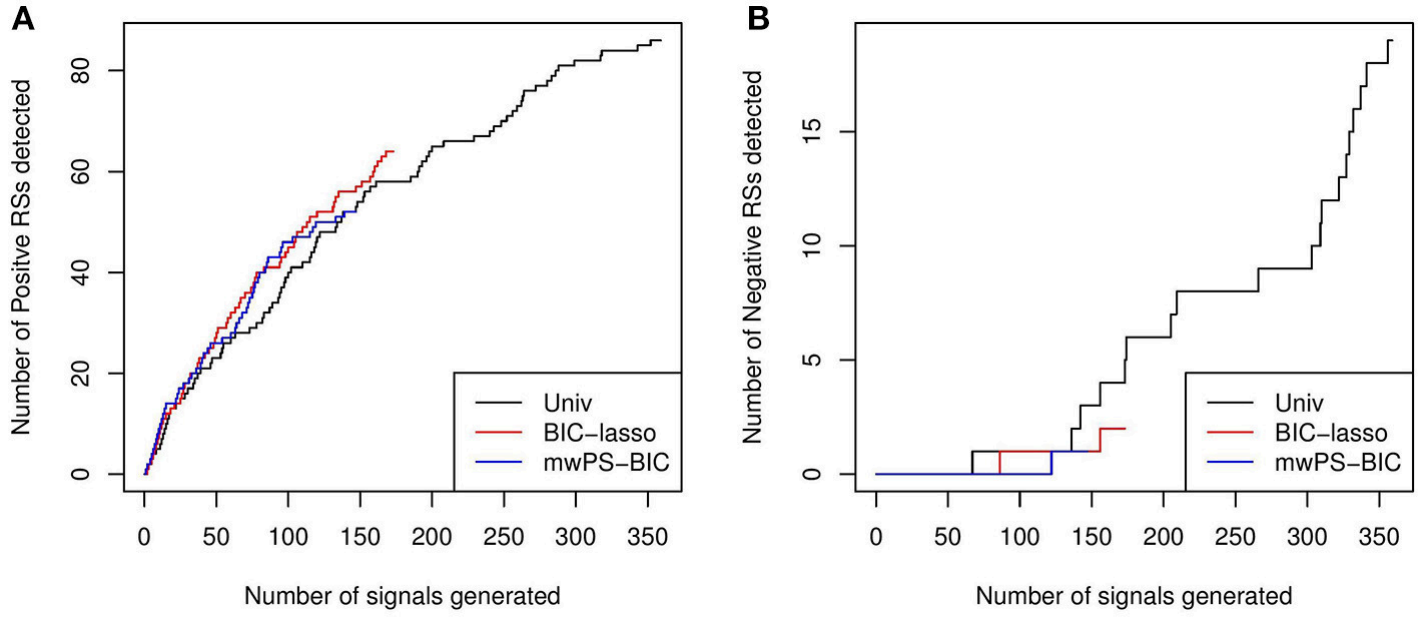
**(A)** Number of positive reference signals detected according to number of signals generated by BIC-Lasso, mwPS-BIC and Univ, where signals are ranked in ascending order by their associated *p*-values for BIC-Lasso and by their adjusted *p*-values for mwPS-BIC and Univ. **(B)** Number of negative reference signals detected according to number of signals generated by BIC-lasso, mwPS-BIC and Univ, where signals are ranked in ascending order by their associated *p*-values for BIC-Lasso and by their adjusted *p*-values for mwPS-BIC and Univ.

Figure 1 shows the number of true positives or true negatives according to the number of signals generated by Univ, BIC-Lasso and mwPS-BIC, where signals are ranked according to their *p*-values for BIC-Lasso, and according to their adjusted *p*-values for Univ and mwPS-BIC. BIC-Lasso and mwPS-BIC were chosen to be represented here since they showed either better or similar performances to the other multiple regression or PS-based approaches respectively (Figures S1–S3). Regarding true positive signal detection, BIC-Lasso performed the best and Univ the worst, mwPS-BIC having a comparable performance to BIC-Lasso. Concerning false positive signal detection, mwPS-BIC performed the best since it detected the first false positive the latest. Univ detected far more signals than mwPS-BIC and BIC-Lasso, but its true positive detection rate was worse on its latest detected signals. Indeed, 36% of its first 150 generated signals were true positive controls compared to 15% of its last 209 signals. The same trend was observed for false positive detection: Univ detected 2% of false positives within its first 150 generated signals and about 7% over its last 209 signals.

Looking more specifically at the PS-based approaches, they behaved similarly within a given PS-estimation method (Figures S1, S2). Considering the three PS methodologies, mwPS-based approaches performed better for an equal number of signals generated (Figure S3).

**Figure 2:**
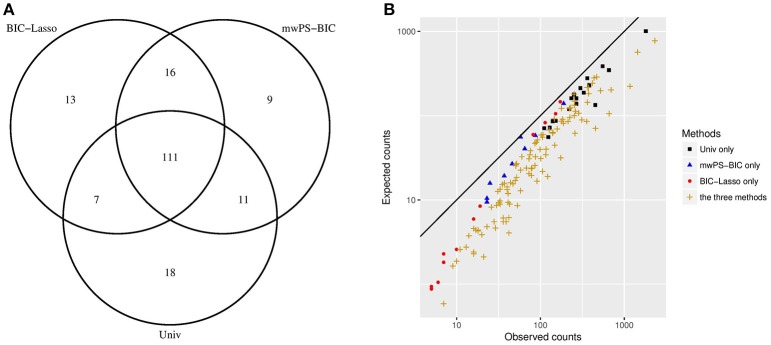
**(A)** Distribution of the first 147 signals generated between Univ, BIC-Lasso and mwPS-BIC. **(B)** Observed counts vs. expected counts of signals generated by Univ only, BIC-Lasso only, mwPS-BIC only and by the three methods, considering their first 147 generated signals. Observed counts *n* are number of reports which involved the signal considered, expected counts *e* are those expected if independence applies between the drug and the AE that form the signal considered . They are calculated as follows: for a signal (drug_*i*_, AE_*j*_) $e_{i,j}=\frac{N_{i}\times N_{j}}{N}$ where *N*_*i*_, *N*_*j*_ are the observed counts of drug_*i*_ and AE_*j*_ respectively, and *N* the total number of observations.

Figure 2A shows the overlap between the first 147 signals generated by Univ, BIC-Lasso and mwPS-BIC and Figure 2B the observed counts *n* vs. the expected counts *e* of some of these signals according to the method(s) by which they were detected. 111 signals were detected by all methods: Figure 2B shows that these signals are characterized by a greater observed risk ratio ($\frac{n}{e}$) and/or a greater support of the data (large *n*) in comparison to signals detected solely by one method. Figure 2B also shows that the methods tend to focus on different types of signals: in particular the BIC-Lasso highlighted some signals with relatively weak support of the data (*n* < 10) whereas Univ highlighted signals with low observed risk but a large number of reports. mwPS-BIC had an intermediary behavior.

## 5. Discussion

Development of novel signal detection methods is crucial to improve the responsiveness and the efficiency of post-marketing surveillance systems. The main issue is to develop techniques that give a reasonable number of signals for further analysis by experts, while providing a list of relevant signals with the fewest possible false associations. Using spontaneous reporting databases is difficult because of their high dimension and extreme imbalance. This study compared recently proposed multiple approaches and a set of methods based on the PS in a signal detection framework. To do so, we used a recently developed set of reference signals pertaining to liver injury: the DILIrank set.

The best performing PS-based approach is the one which relies on the MW. Its performance in terms of true/false signal detection, specificity and sensitivity is very close to that of the multiple regression approaches, which performs the best. It also generates roughly the same number of signals as BIC-Lasso and CISL, the latter being conservative for highly reported adverse events, as is the case for DILI (Ahmed et al., 2018). The mwPS-based approaches have an intermediary behavior between multiple regression approaches and univariate approach in the type of signal generated.

In accordance with the literature, adjustment on the PS does not achieve the best performances (Stuart, 2010). The adjustment approaches had similar behavior to the univariate approach. In particular, they generated very large numbers of signals in comparison to the other PS-based approaches and multiple regression approaches, and they had poor specificity and good sensitivity.

The iptwPS-based approaches performed extremely poorly. Unlike the MW, weights from IPTW are not normalized and could be very large for untreated individuals with low PS. This numerical instability due to high weights with IPTW weighting has already been reported (Yoshida et al., 2017). To avoid this issue, a solution is to do weight-trimming: weights greater than a given value are assigned this value (Seeger et al., 2017).

In addition to the well-known variable selection algorithm in PS estimation, hdPS, here we implemented three other PS-estimation methods: two based on variable selection algorithms, BIC-Lasso or CISL, and one derived from a machine-learning algorithm: GTB. A drawback with the hdPS algorithm in our setting is that the PS obtained with this method is AE dependent: for each drug to be included in the PS, its association with the AE under study has to be computed. This can become very time-consuming when screening several thousands of AEs. On the contrary, the three other PS-estimation methods have a computational advantage: once the PS is estimated for a given drug, it can be used to test associations with any AEs. When PS is estimated with the covariates selected by BIC-Lasso, CISL or with a GTB algorithm, PS-based approaches are competitive with multiple regression approaches in terms of calculation time. Regressions like weighted univariate logistic regressions can be easily performed. Depending on the PS methodology used (adjustment or weighting), results are roughly comparable according to the way PS is estimated.

A major constraint in developing signal detection methods is to obtain a reliable set of reference signals to assess performances. Here we used a recently established set, the DILIrank set pertaining to a common adverse event, which is not the case for all the AEs in the database. Furthermore, three levels of DILI risk assessment are defined in DILIrank for drugs: most DILI concern, less DILI concern and no DILI concern. We chose to consider only most DILI concern drugs as true positive controls, leaving less DILI concern drugs, because we decided to focus on drugs which could lead to severe DILI related outcomes.

The results suggest that the proposed PS-based methodology is an interesting complement to other existing methods. In a sense, it combines the main strengths of both univariate and multiple regression approaches: it makes it possible to account for co-reported drugs while using multiple hypothesis testing theory as regards the detection threshold. Further studies on alternative reference sets and simulation studies will be useful to confirm its potential for automated signal detection in pharmacovigilance.

## Author contributions

ÉC, IA, and PT-B conceived and designed the study. ÉV contributed to the design of the work. AP and FS contributed to the acquisition and the interpretation of data for the work. ÉC performed the computations. ÉC, IA, PT-B, and ÉV discussed the results. ÉC drafted the manuscript with support from IA and PT-B. All authors critically revised the work and approved the final manuscript.

### Conflict of interest statement

The authors declare that the research was conducted in the absence of any commercial or financial relationships that could be construed as a potential conflict of interest.
