# Supplementary material for: Propensity Score-Based Approaches in High Dimension for Pharmacovigilance Signal Detection: an Empirical Comparison on the French Spontaneous Reporting Database
Source: Front Pharmacol. 2018 Sep 18;9:1010. doi: 10.3389/fphar.2018.01010 (PMC6153352; doi:10.3389/fphar.2018.01010)
Supplement: Supplementary file 1 [file Presentation_1.pdf]

Supplementary Material:
Propensity score-based approaches in high dimension for pharmacovigilance signal detection: an empirical comparison on the French spontaneous reporting database

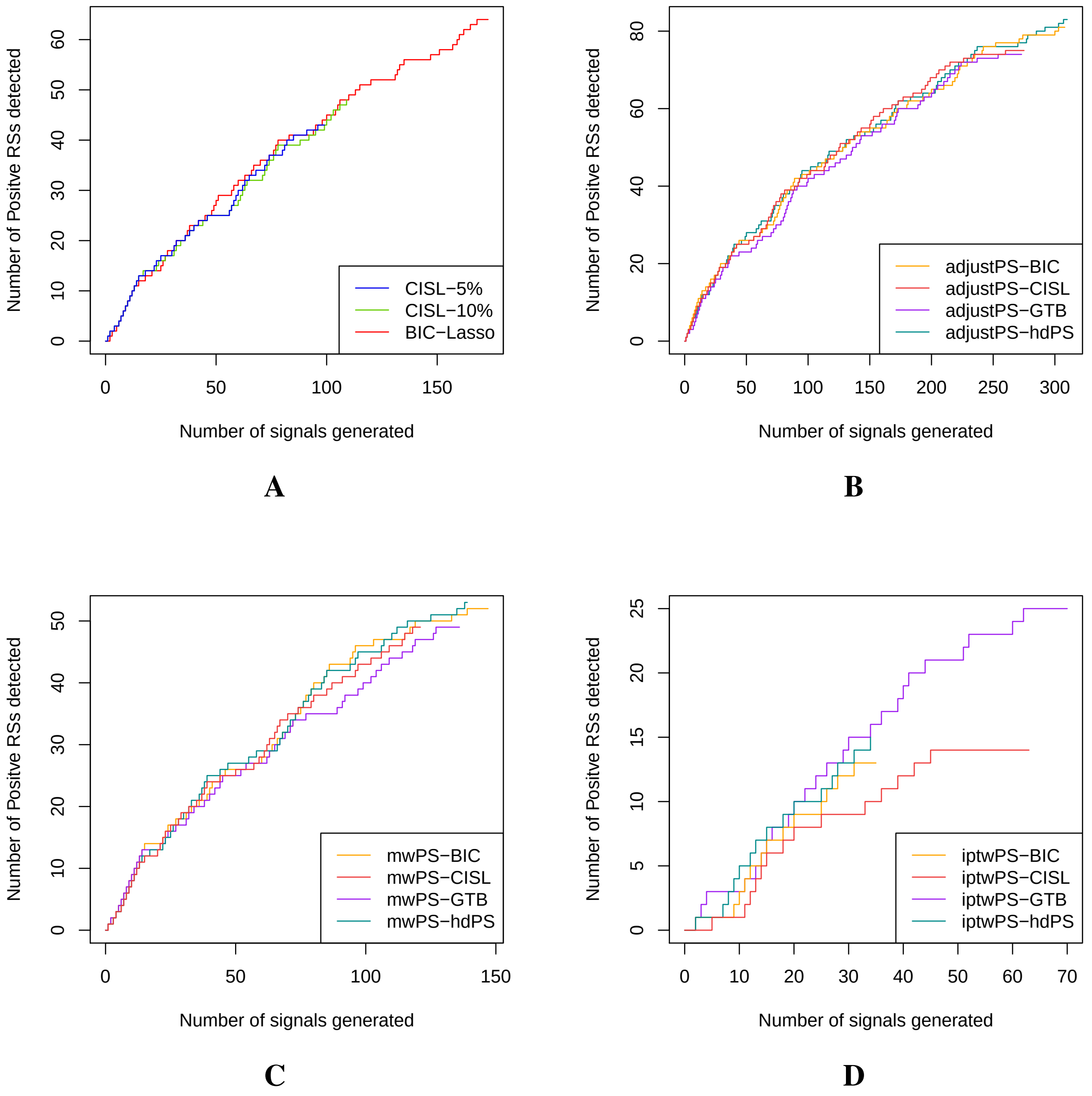

Figure S1: **(A):** Number of positive reference signals detected according to number of signals generated by multiple regression approaches: BIC-lasso, CISL-5% and CISL-10%, where signals are ranked in ascending order by their associated p-values for BIC-Lasso, and in decreasing order of their 5% or 10% output distribution quantile value for CISL-5% and CISL-10%. **(B):** Number of positive reference signals detected according to number of signals generated by adjustment on propensity score methods, depending on how the propensity score was estimated: adjustPS-BIC, adjustPS-CISL, adjustPS-GTB and adjustPS-hdPS, where signals are ranked in ascending order by their adjusted p-values. **(C):** Number of positive reference signals detected according to number of signals generated by weighting with matching weights on propensity score methods, depending on how the propensity was estimated: mwPS-BIC, mwPS-CISL, mwPS-GTB and mwPS-hdPS, where signals are ranked in ascending order by their adjusted p-values. **(D):** Number of positive reference signals detected according to number of signals generated by inverse probability of treatment weighting on propensity score methods, depending on how the propensity was estimated: iptwPS-BIC, iptwPS-CISL, iptwPS-GTB and iptwPS-hdPS, where signals are ranked in ascending order by their adjusted p-values.

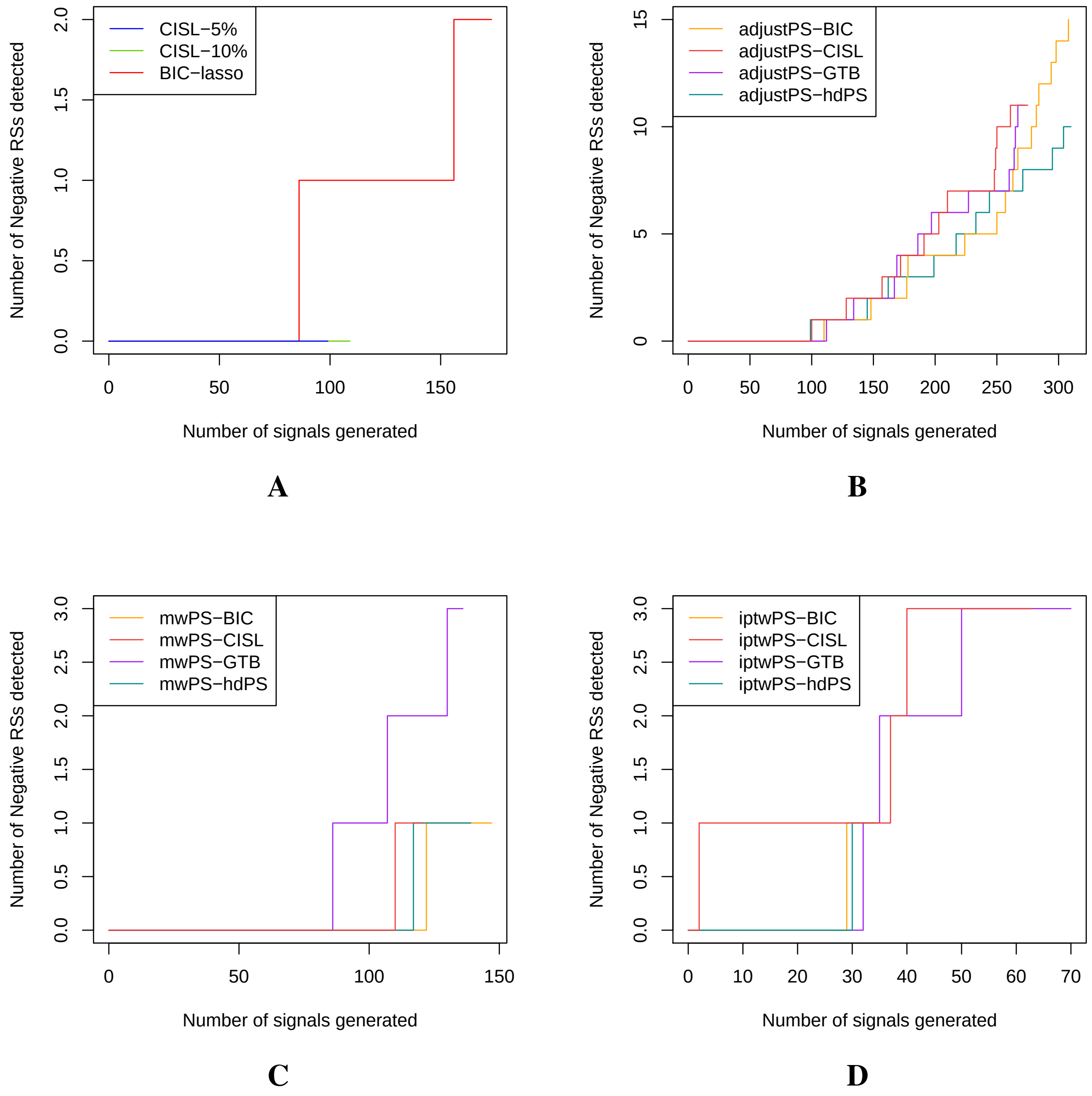

Figure S2: **(A):** Number of negative reference signals detected according to number of signals generated by multiple regression approaches: BIC-lasso, CISL-5% and CISL-10%, where signals are ranked in ascending order by their associated p-values for BIC-Lasso, and in decreasing order of their 5% or 10% output distribution quantile value for CISL-5% and CISL-10%. **(B):** Number of negative reference signals detected according to number of signals generated by adjustment on propensity score methods, depending on how the propensity score was estimated: adjustPS-BIC, adjustPS-CISL, adjustPS-GTB and adjustPS-hdPS, where signals are ranked in ascending order by their adjusted p-values. **(C):** Number of negative reference signals detected according to number of signals generated by weighting with matching weights on propensity score methods, depending on how the propensity was estimated: mwPS-BIC, mwPS-CISL, mwPS-GTB and mwPS-hdPS, where signals are ranked in ascending order by their adjusted p-values. **(D):** Number of negative reference signals detected according to number of signals generated by inverse probability of treatment weighting on propensity score methods, depending on how the propensity was estimated: iptwPS-BIC, iptwPS-CISL, iptwPS-GTB and iptwPS-hdPS, where signals are ranked in ascending order by their adjusted p-values.

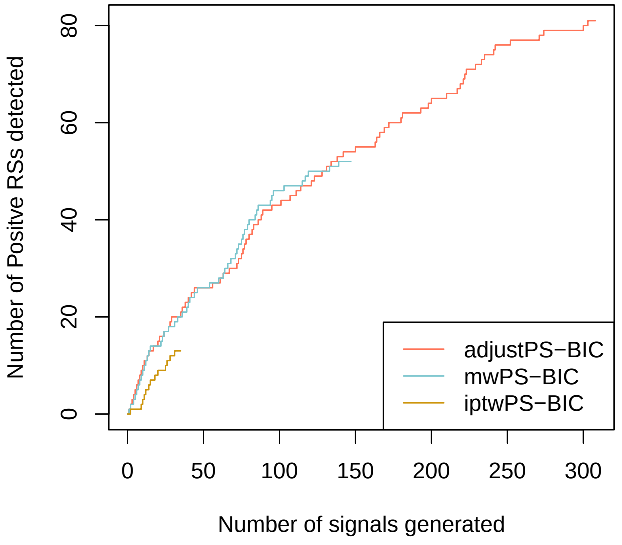

A

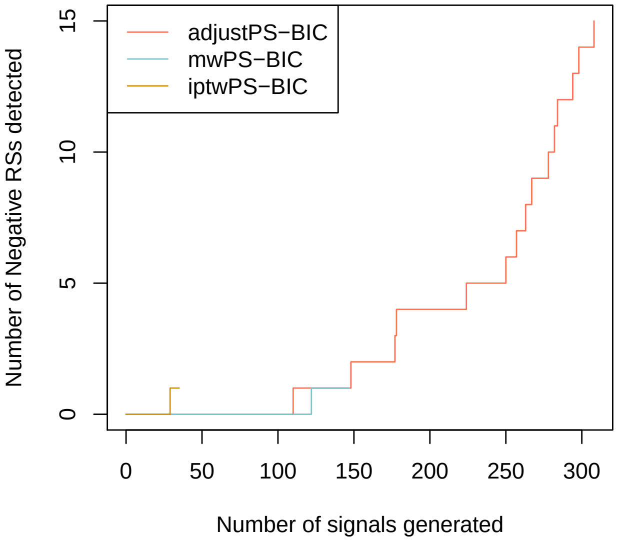

B

Figure S3: **(A):** Number of positive reference signals detected according to number of signals generated by adjustment on propensity score methods, weighting with matching weights on propensity score methods and inverse probability of treatment weighting on propensity score methods, where the score is estimated with BIC-Lasso methodology: adjustPS-BIC, mwPS-BIC, iptwPS-BIC, where signals are ranked in ascending order by their adjusted p-values. **(B):** Number of negative reference signals detected according to number of signals generated by adjustment on propensity score methods, weighting with matching weights on propensity score methods and inverse probability of treatment weighting on propensity score methods, where the score is estimated with BIC-Lasso methodology: adjustPS-BIC, mwPS-BIC, iptwPS-BIC, where signals are ranked in ascending order by their adjusted p-values.
